# How does the public understand the causes of mental disorders? An analysis of Irish news media before and during the COVID-19 pandemic

**DOI:** 10.1371/journal.pone.0284095

**Published:** 2023-04-06

**Authors:** Leigh Huggard, Cliódhna O’Connor

**Affiliations:** School of Psychology, University College Dublin, Belfield, Dublin 4, Ireland; Regional Health Care and Social Agency of Lodi, ITALY

## Abstract

Public perceptions of the determinants of mental illness have important implications for attitudes and stigma, but minimal previous research has explored how causal attributions are spontaneously invoked in everyday public discourse. This study investigated how causal explanations for mental illness are disseminated in popular Irish news media, in the two years before and after the onset of the COVID-19 pandemic. Keyword searches of a news media database identified 1,892 articles published between March 2018 to March 2022 that mentioned one of six categories of mental disorders: anxiety disorders, mood disorders, substance-related disorders, personality disorders, eating disorders, and psychotic disorders. Overall, 25% of the identified articles contained a causal explanation for mental illness. Inductive content analysis revealed the content and prevalence of eight types of causal explanations for mental disorders. Overall, attributions to life events/experiences, the cultural/societal environment, interpersonal relations, and health and lifestyle factors occurred more frequently than attributions to biological or psychological determinants. Life events/experiences were the most common explanation for anxiety and personality disorders, cultural/societal environment for eating disorders, and health/lifestyle factors for mood and psychotic disorders. Interpersonal factors in mental illness aetiology became more salient following the COVID-19 pandemic. The findings emphasise the need for theory and research on lay explanations of mental disorders to account for diversity, both in the range of attributions invoked, and in how attributional patterns shift across time and mental disorders category.

## Introduction

Public beliefs about the causes of mental illness have important implications for people with mental health difficulties. For instance, the belief that biological factors are responsible for mental illness has been shown to be associated with greater stigmatising attitudes, such as desire for social distance or perceptions of dangerousness [1, 2]. Exposure to information about mental illness that circulates in public discourse, such as through news media, can sway public opinion [3], and in the past has contributed to the formation of negative attitudes and harmful stereotypes [4]. Exploring how causal explanations are disseminated in real-world contexts can provide insight into how members of the public encounter such information, and consequently, how their understandings may be shaped. The current paper reports a content analysis of causal explanations for mental disorders in Irish news media, which provides first evidence of the variety of attributions for mental illness offered in public discourse, the frequency with which they occur, and their variability across disorder category and time.

### The significance of causal attributions for mental illness

In societies with high mental health literacy, causal explanations are a common discussion-point in conversations about mental health [5]. As proposed by attribution theory [6], this may be due to a natural tendency to search for meaning behind unusual or challenging phenomena [7]. Causal attributions have been shown to aid people with mental health difficulties in navigating their own experiences with mental illness by redirecting blame away from themselves [8], and finding meaning in their experiences [9]. Conversely, attributions that foster self-blame can impede adjustment and coping processes [10].

Moreover, the causal attributions of the general public are key influences on attitudes to and treatment of people with mental illness. In recent decades, public campaigns aimed at reducing stigma or increasing mental health literacy have promoted a medical model of mental illness, often citing genetics or brain chemistry as responsible [2, 11, 12]. However, there is clear evidence to suggest that diverse social factors are also responsible for mental illness, including experiencing or being the subject of violence, discrimination, socioeconomic disadvantage, poor social cohesion, and more [13]. Research has suggested that members of the public underestimate the social determinants of physical illness [14], but it is unknown whether similar patterns exist for mental illness. While mental health literacy research has suggested the public often favour psychosocial attributions for mental illness [5], there is evidence that biogenetic attributions are becoming more commonplace [12, 15, 16]. Yet the longitudinal rise in biogenetic attributions has not been paralleled by observed improvements in stigmatising attitudes towards people experiencing mental health difficulties, and in the case of certain mental illnesses such as schizophrenia, stigma may be increasing [12, 17, 18].

Empirical research has shown that the medicalisation of mental illness can promote unfavourable attitudes towards people with mental health difficulties. Extensive meta-analytic evidence has found that while biological attributions for mental illness are associated with reduced blame towards people with mental illness, they are also associated with greater perceived dangerousness, greater desire for social distance, and greater prognostic pessimism than social attributions for mental illness [1, 2]. This may be explained by theories of psychological essentialism, which suggest that biogenetic explanations promote the lay belief that underlying “essences” are responsible for a group’s shared characteristics, which in turn triggers the aforementioned stigma attitudes [1]. Indeed, Dittrich et al. [19] report that essentialism fully mediates the relationship between biogenetic explanations of schizophrenia and increased social distance. The relationships between biological attributions and negative social outcomes have been observed in both cross-sectional [20] and experimental [21] research, with the latter demonstrating that causal beliefs can be manipulated by exposure to experimentally-presented causal explanations.

While extensive research has investigated how biological explanations may trigger essentialist ways of thinking, much less evidence illuminates the consequences of social attributions for mental illness. The majority of previous studies have used a brief generalised measure of “social explanations” as a control or comparative point for biological explanations [21]. Minimal research has investigated the potentially variable consequences of endorsing different types of social determinants of mental illness. Social psychologists studying other forms of intergroup relations have proposed the existence of a social component of essentialism, whereby certain social explanations may be associated with similar levels of essentialist thinking as biological explanations [22]. In particular, social attributions perceived as being highly deterministic (e.g., one’s upbringing or neighbourhood environment) may be associated with a greater degree of essentialist thinking than social attributions perceived as being more malleable (e.g., a recent traumatic experience, such as a car crash). For example, people with deterministic beliefs about one’s social origin show more prejudice against other nationalities [22]. It is unknown whether different social attributions for mental illness have distinct attitudinal consequences. Even more fundamentally, no research has catalogued the range and relative prevalence of social explanations for mental illness, which circulate in the public sphere.

### Variations in causal attribution patterns

A nuanced understanding of lay causal attributions for mental illness requires attention to how attributional patterns can vary across context. Beyond a tendency to collapse potentially diverse social attributions into a single unidimensional category, much previous literature on lay attributions for mental illness is also limited by a tendency to conceptualise ‘mental illness’ as one universal category [23]. This masks any potential variation in attitudes across specific diagnoses [24]. There is some evidence that members of the public hold differing causal beliefs across mental disorder type. For instance, a review by Angermeyer and Dietrich [23] found that people are more likely to endorse biological factors as being responsible for schizophrenia than for depression. Moreover, the repercussions of certain attributions can differ across disorder categories: for example, a recent study of lay attitudes to functional (psychogenic non-epileptic/dissociative) seizures links biomedical explanations with less negative attitudes [25]. Observing differences in causal representations across different types of mental disorders can thus provide crucial nuance that would otherwise be lost in studying causal explanations of a generalised concept of mental illness alone.

As well as varying across mental disorder type, causal attributions are known to vary across time and culture [2, 5, 26]. For instance, in western cultures, as biological attributions have risen in popularity over recent decades, attributions to supernatural factors have dropped significantly [2, 12]. How aetiology is conceptualised is also contingent on historical context. An experimental study investigated how the COVID-19 pandemic reshaped public beliefs about the causes of mental disorders, finding that when people read about a specific (fictional) case of anxiety symptoms that began during the pandemic, generalised attributions to biological causes weakened [27] However, such experimental designs necessarily rely on contrived scenarios that require participants to respond to predefined scenarios in predefined ways. Full understanding of the malleability and contextual contingency of lay explanations for mental disorders requires an ability to track the causal attributions that spontaneously occur in everyday discourse, across particular points in time.

### Media representations and their implications

An ecologically valid exploration of attributional patterns in public discourse, which can incorporate attention to time and context, necessitates access to naturally-occurring data. The mass media are a key site at which lay publics are exposed to aetiological explanations for mental disorders. While media content does not fully determine lay understandings, it nevertheless plays an important role in shaping public opinion [28]. This is particularly the case regarding more abstract or scientific topics, such as the aetiology of mental disorders, which are not perceptible to immediate observation or experience [29, 30]. Media data offer a naturalistic opportunity to establish the frequency with which causal attributions spontaneously appear, and the range of causes that are invoked, within everyday discourse about mental disorders.

The present study uses the Republic of Ireland as a case study to spearhead an original study of naturalistic attributions for mental disorders in public discourse. The history of mental illness in Ireland is characterised by stigmatisation and incarceration, gradually ceding to increasing mental health awareness and therapeutic support [31]. However, mental health provision in Ireland remains severely under-resourced, with long waiting-lists and minimal specialist services. An OECD report [32] found Ireland had the 3^rd^ highest prevalence of mental illness in Europe, with nearly one-in-five residents qualifying for a psychiatric diagnosis. Demand for mental health services, and the salience of mental illness as a public health issue, appear to have increased since the COVID-19 pandemic [33], making this a timely context to appraise how public understandings of mental disorders are evolving.

This study uses content analysis to identify the causal explanations for various mental disorders, and their prevalence, in major print and online news media in Ireland. The paper builds on previous literature by revealing the ways in which the public are exposed to causal explanations of mental disorders, paying particular attention to representations of social determinants, which are under-researched in previous literature. It also illuminates differences in news representations across mental disorders type by comparing across six categories of mental disorders. Finally, it investigates the malleability of attributions by exploring whether they shifted following the onset of the COVID-19 pandemic. In line with a ‘cultural meanings’ rather than ‘deficit model’ approach to lay understandings [34], the concern is not with evaluating the accuracy or ‘truth’ of public attributions, but documenting their range, prevalence, and distribution across time and mental disorder category.

### Primary research question

- How are causal explanations for mental disorders represented in Irish news media?

### Secondary research questions

- What proportion of articles that mention mental disorders contain causal explanations?
- Are causal explanations represented differently across different categories of mental disorders?
- Did patterns of causal explanations change during the COVID-19 pandemic?

## Methods

### Data collection

News articles were identified using the Nexis Advance database. Search terms were devised based on DSM-5 diagnostic terms and categorisations [35; S1 Appendix]. Six categories of mental disorders were chosen based on global prevalence rates [35, 36]: anxiety disorders, feeding and eating disorders, mood disorders (bipolar and related disorders and depressive disorders), schizophrenia spectrum and other psychotic disorders, personality disorders, and substance-related disorders. Searches were limited to Irish national news articles between 12^th^ March 2018 and 12^th^ March 2022.All national news sources available through Nexis Advance were included, encompassing 11 online and print news sources (S2 Table). Local newspapers were excluded. Articles identified from the search strings (*n* = 1,892) were downloaded to NVivo for content analysis [37].

### Data analysis

Articles were categorised based on mental disorder category and whether they were published before or after the beginning of the COVID-19 pandemic. The 12^th^ March 2020 was chosen to denote the beginning of the pandemic, as on this date the Irish Taoiseach (Prime Minister) issued an official announcement formally acknowledging the COVID-19 crisis as a pandemic and introducing measures to tackle the virus.

All articles were read through to identify any statement indicating a causal explanation of a mental disorder, defined as any explicit or implied reasoning for the onset/progression of the mental disorders in question. Inductive content analysis was used to identify and classify different types of causal explanations. A preliminary coding frame was developed based on the analysis of 400 randomly-chosen articles covering all of the six mental disorder categories. Similar attributions were grouped into basic codes (e.g., social media/the internet), which were then organised into overarching superordinate code categories (e.g., cultural/societal environment; S1 Table). For context, each attribution was also coded to denote its source (i.e., the type of person/organisation who offered it).

Relevant codes were attached to each article using NVivo. Codes were not treated as mutually exclusive, and where relevant, explanations presented in the articles were assigned one or more codes. All articles were coded by reviewer one (L.H.), and approximately one-third of the coded articles (*n* = 178) were additionally independently coded by reviewer two (C.O.C.). Inter-coder agreement [38] was calculated using the coding comparison function in NVivo. Overall agreement was 92.8% (*k* = .874; S1 Table). The coding frame was then applied to the remaining articles and slightly modified as appropriate. A frequency analysis was conducted using the Matrix function in NVivo.

### Ethics statement

Since all data were publicly available and written with the intent of public consumption, ethical approval was not required for this analysis.

## Results

### Overall prevalence of causal explanations

**Fig 1:**
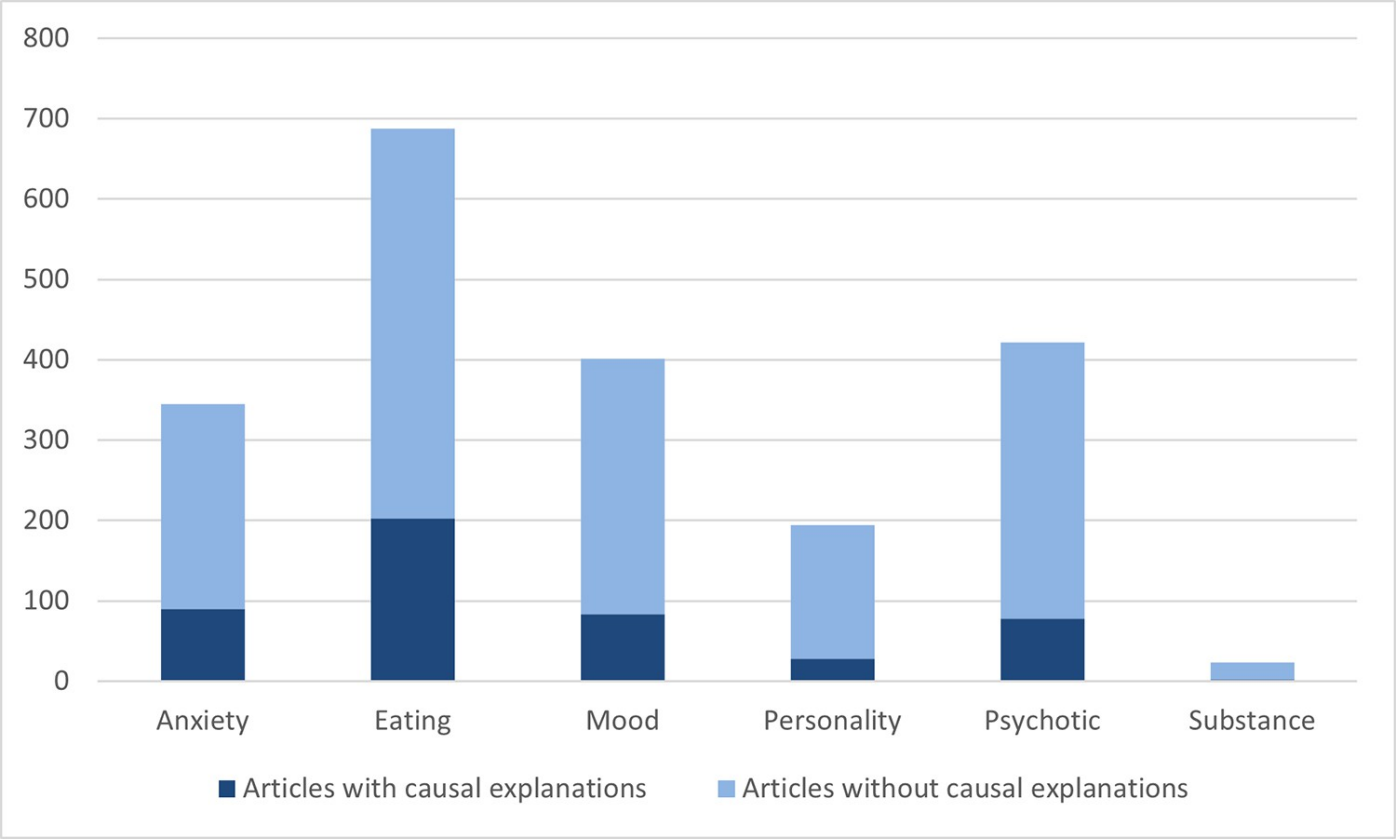
Frequencies of articles with and without causal explanations.

Overall, 24.63% (*n* = 466) of the identified articles contained causal explanations for a mental disorder (Fig 1). Due to many of the articles containing multiple attributions, the total number of causal explanations came to a total of 628. Frequencies of articles containing causal explanations varied across type of mental disorder, from 8.70% for substance related disorders, to 29.50% for eating disorders.

In terms of the source of attributions, most explanations (51%) originated from an individual’s perception of their own experience with mental illness, whether directly relayed by themselves or someone else. Other sources included relevant experts, such as doctors, psychologists, and scientists (19%), scientific research (17%), and other origins (7%) such as political figures or commentators presented as knowledgeable on the topic.

### Categories of causal attributions

**Fig 2:**
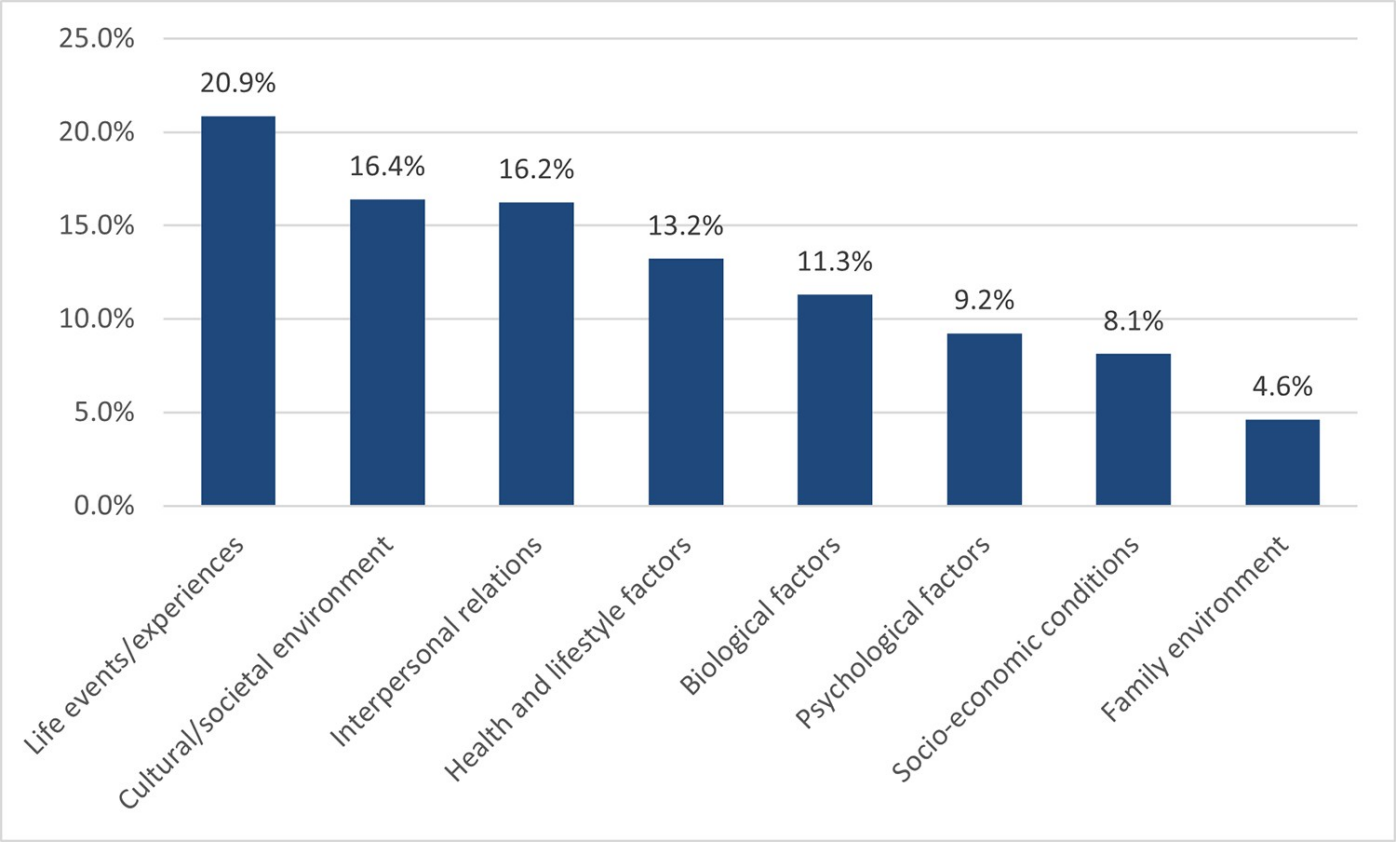
Frequency of different causal attributions for mental disorders present in Irish news media. Frequencies represent the number of attributions in a given category as a percentage of the total number of attributions.

Using content analysis [37], eight superordinate attributions for mental illness were identified, and their frequencies were calculated as a percentage of the total number of attributions overall (*n =* 628; Fig 2). These were: life events/experiences (20.86%, *n* = 131), the cultural/societal environment (16.40%, *n* = 103), interpersonal relations (16.24%, *n* = 102), health and lifestyle factors (13.22%, *n* = 83), biological factors (11.30%, *n* = 71), psychological factors (9.24%, *n* = 58), socio-economic conditions (8.12%, *n* = 51), and the family environment (4.62%, *n* = 29).

#### Life events/experiences

Significant life events or experiences appeared as the most frequent type of causal explanation for mental disorders (20.86%, *n =* 131).

Traumatic experiences and adversity were seen as inducing mental disorders in many of the coded articles (*n* = 81). This included experiences such as sexual assault, motor accidents, losing a loved one, and incidences of abuse, among others.

*…the now 19-year-old woman said the attacks have left her suffering from post traumatic stress disorder. She said she dropped out of school and developed anorexia*.

*(Irish Independent, 21/09/2021)*

Another life experience to which mental disorders was frequently attributed was losing a loved one (*n* = 16).

*…my sister Robin had been killed in a car crash. My mother quickly descended into paranoid schizophrenia*.

*(Irish Daily Mail, 31/03/2018)*

Life experiences and situations that were stress-inducing were seen as responsible for mental disorders (*n* = 12).

*In her 20s, early success, on top of the usual stresses of being a young woman, threatened to overwhelm her, and Collette developed bulimia and suffered panic attacks. In retrospect, she thinks there was just a lot of change going on in her life, perhaps too much*.

*(Sunday Independent, 24/06/2018)*

Pressure relating to one’s education, such as exam stress or school-related expectations were common specific examples (*n* = 13).

*There was a lot of pressure for exams. For me, the anxiety started getting much worse. I couldn’t control my brain to learn in that rote way, so this was my way of controlling something small. I wasn’t happy with my body*.

*(Sunday Independent, 20/01/2019)*

The experience of childbirth or pregnancy, or negative postpartum events/experiences was also referenced as a mental illness determinant (*n* = 5).

*His mother developed postpartum psychosis following his birth more than 50 years ago and went on to suffer schizophrenia for the rest of her life*.

*(The Irish Times, 21/10/2020)*

Some articles also included generic mentions of “environmental factors”, presumably representing life experience generally, as being responsible for mental disorders (*n* = 6).

*Schizophrenia and other psychotic conditions are now known to arise from a complex interplay of genes and environment…*

*(Irish Daily Mail, 19/03/2019)*

#### The cultural/societal environment

Aspects of one’s social environment were also commonly described as being responsible for mental disorders (16.40%, *n* = 103).

Cultural/societal expectations were one example of this (*n* = 56). This included perceived pressure from those around oneself to appear or behave in certain ways, including gender expectations, education and employment pressures, expectations around physical appearance and fitness, and expectations associated with fame and being in the public eye.

*"Our cultural obsession with weight and diets is having a hugely detrimental impact on women’s emotional health, particularly young women,"*

*(Irish Independent, 24/10/2019)*

Another component of one’s cultural or societal environment that was positioned as negatively impacting mental health was social media and the internet (*n* = 32), often described as exacerbating negative societal expectations such as beauty standards or fitness standards, as fostering smartphone addiction and impulsivity, or as facilitating access to websites encouraging eating disorders.

*…she has spotted a trend of people presenting with depression and anxiety but once she scratches beyond the surface, she sees unhealthy fixations with mobile smartphones. "Some people can’t leave it behind them, they’re constantly on 24–7 and this affects their mental health,"…*

*(Irish Independent, 02/01/2019)*

Overexposure to fear-inducing or distressing information was also a factor deemed responsible for both causing and exacerbating the symptoms of mental illness (*n* = 11). This information commonly centred around topics such as climate change, COVID-19, animal cruelty, and other health and wellness related information.

*His breakdown, when it came nearly half a century later, would be triggered by news reports of cruelty to animals in Africa, particularly elephants, that left him feeling angry and powerless*.

*(Irish Daily Mail, 12/03/2021)*

Amongst the less common features of the cultural or societal environment attributed to influencing mental disorders were modern ways of living, rapid cultural change, political events, and a lack of coherency between the individual and their societal environment (*n* = 9).

*A lot of our anxiety is because we don’t live in a natural way any more. We are detached from ourselves as animals. There is no separation between day and night. We sleep less and sleep worse because of the blue lights on our phones beside our beds, watching Netflix late at night, etc…*

*(The Irish Times, 09/03/2019)*

#### Interpersonal relations

Analysis of the news articles revealed that one’s relationships and interactions with those around them was represented as an important determinant of mental health difficulties (16.24%, *n* = 102).

Social isolation and loneliness, particularly associated with COVID-19 lockdowns, was positioned as one aspect of this (*n* = 50).

*Aware, a charity which provides assistance for those affected by depression, bipolar disorder, and other moodrelated conditions, said it had seen a "dramatic increase" in demand for its services since lockdown began in March*.

*(Irish Independent, 16/11/2020)*

The mistreatment of an individual by those around them was also positioned as responsible for mental health problems (*n* = 45), including in the forms of bullying, harassment, discrimination, and body shaming, among others.

*"I was bullied in transition year and fifth year” … The eating disorder, unfortunately, was a coping mechanism*.

*(Irish Independent, 15/03/2021)*

Other features of interpersonal interactions to which mental disorders were attributed included general relationship issues, being compared to other people, and contact with other people with mental illness (*n* = 7).

*The problem—one of them, anyway—is that anxiety is infectious. "The thing about anxiety is, if I’m anxious about something, it’s my primal urge to make you anxious,"*

*(Sunday Independent, 31/03/2019)*

#### Health and lifestyle factors

Other types of causal explanations for mental disorder in the identified articles included various health and lifestyle factors (13.22%, *n* = 83).

This included some medications and substances, including taking both prescribed and non-prescribed medication, substance withdrawal, the side-effects of medications, alcohol, nicotine, and recreational drugs (*n* = 38).

*Previous research tells us that young people who use cannabis frequently have worse outcomes in life than their peers and are at increased risk for serious mental illnesses like schizophrenia*

*(The Irish Times, 20/01/2021)*

Diet, including malnourishment, vitamin deficiency, weight loss diets, and limiting variety or magnitude of food intake was also referenced (*n* = 17).

*While there was little evidence that fatty acids were associated with mental disorders at age 17, the researchers found that 24 year olds with psychotic disorder, depressive disorder and generalised anxiety disorder had higher levels of omega-6 than omega-3 fatty acids compared to those without these disorders*.

*(Irish Daily Mail, 08/06/2021)*

Causal explanations also included a person’s sleeping pattern or the amount of sleep they received (*n* = 13).

*If you live against your natural rhythms—for example, your body wants you to go to sleep at 9pm, but you force yourself to stay up later—you are at high risk for sleep deprivation, chronic stress, mood disorders, lowered immunity and compromised overall health*.

*(Irish Daily Mail, 08/01/2022)*

Physical illnesses or health issues were also identified as determinants of mental illness (*n* = 7).

*An Oxford study published in the Lancet Psychiatry journal last April looked at the records of almost a quarter-of-a-million patients diagnosed with Covid-19: a third had a neurological or psychiatric diagnosis in the six months following their Covid-19 diagnosis*

*(Sunday Independent, 17/20/2021)*

Other health and lifestyle factors included a lack of physical activity, changes to one’s daily routine, and exposure to pollution (*n* = 8).

*Data from Denmark showed those growing up in the most polluted areas had a rate of schizophrenia almost 1.5 times higher than those in the least polluted areas*.

*(Irish Daily Mail, 2019)*

#### Biological factors

Biological factors accounted for 11.30% (*n* = 71) of the causal attributions for mental disorders present in the analysed articles. These were most commonly represented as heritable factors, but other biological attributions included brain injury, hormones and neurotransmitters, and more.

Genetic explanations described mental disorders being passed down from family members (*n* = 41).

*… decades of twin studies have helped to establish that anorexia nervosa is 50–60 per cent heritable*.

*(The Irish Times, 02/04/2019)*

Several types of hormones and neurotransmitters were identified as determinants of mental disorder (*n* = 10), with descriptions ranging from general mentions of “brain chemistry” to identifying specific chemicals such as oestrogen or serotonin.

*Quite why people develop bipolar disorder is not clear cut. In some cases, it is thought brain chemistry may be to blame*

*(Irish Daily Mail, 15/12/2020)*

Injuries to the brain, including stroke and head trauma, were also described as responsible for mental illness (*n* = 3).

*…she said that while the family had suffered tragedy, it was down to bad luck and not inherited mental disorders. Her own mother was told she had schizophrenia by doctors who were unaware that she had suffered ’a massive brain injury’ when she was run over in 1929*

*(Irish Daily Mail, 09/01/2021)*

Other biological factors included menopause, enzymes, and pre-natal or intra-uterine factors (*n* = 22).

*A large-scale Swedish study of 90 per cent of births over 28 years found that children born to fathers over the age of 45 were at greater risk of autism, bipolar disorder, suicidal behaviour, drug abuse and ADHD*.

*(The Irish Times, 28/07/2018)*

#### Psychological factors

Various psychological factors appeared in the texts as being responsible for mental disorder (9.24%, *n* = 58).

Mismanaging one’s emotions was one of such factors, which included supressing emotions, feeling a lack of control, and other related problems (*n* = 26).

*…the more unwilling we are to feel anxiety, the more likely we are to experience a clinically diagnosable anxiety disorder*.

*(Irish Independent, 28/01/2019)*

Other sources attributed mental disorders to one’s character, personality, or disposition (*n* = 23).

*I have an anxious nature and it is part of me, inextricably knitted into the fibre of my being. Even before I was fully aware of it, it was there, just dormant*.

*(Irish Independent, 28/01/2019)*

Other psychological factors included neurodiversity and identity struggles (*n* = 9).

*According to Laura, individuals on the spectrum are more prone to anxiety because they live in a neurotypical world that really wasn’t built for them, making it a much more confusing place*.

*(Irish Independent, 06/10/2021)*

#### Socio-economic conditions

One-in-twelve (8.12%, *n* = 51) explanations for mental disorders pertained to socio-economic conditions.

In particular, mental disorders were often attributed to employment conditions (*n* = 23), such as working conditions, losing one’s job, overworking, job insecurity, and stresses relating to running a business.

*She was, she reflects now, all about work … In the end, her body essentially gave up. "I got to a point where I had to go to bed. My body was just done." She began experiencing daily panic attacks…*

*(Sunday Independent, 25/08/2019)*

Financial issues and poverty experienced in both childhood and adolescent were identified in the included articles (*n* = 13).

*The year that I stopped displaying any symptoms, when the hellish, almost decade-long cycle of depression, mania, and episodes of anxiety, psychosis and a general feeling of being out-of-control ceased for apparently no reason, I analysed my life to find out what had changed. Only two things had changed: I had stopped dating, and my financial situation had become more secure*.

*(The Irish Times, 02/02/2019)*

Housing and living conditions, including insecure housing, poor living conditions, growing up in foster care, and unstable living conditions also appeared in the identified articles (*n* = 11).

*I have had my fair share of issues, including an eating disorder in my 20s, but I cannot say for certain that their divorce was to blame. Being sent to boarding school at the age of eight was, to me, far more damaging*.

*(Irish Daily Mail, 29/04/2021)*

Other attributions included socio-economic inequalities and general mentions of socio-economic factors (*n* = 6).

*Anxiety rates, generally, are on the increase in wealthier countries. International studies indicate the proportion of Irish teens with the condition is not significantly out of line with other developed countries*.

*(The Irish Times, 26/05/2018)*

#### The family environment

One’s family environment was described as responsible for mental disorders in 4.62% (*n* = 29) of articles containing causal explanations.

Receiving low parental attention or affection was one feature of one’s family environment seen to cause mental disorders (*n* = 8).

*I always overate and while I didn’t admit it at the time, looking back I can see that it was a form of attention seeking as my parents fostered children, so the house was always busy and I probably didn’t feel I was getting enough time from them*

*(Irish Independent, 14/01/2019)*

Having a parent with either physical or mental health issues was another factor described as being associated with mental disorders (*n* = 6).

*Now that I know myself more, I realise there was a lot of stress in my childhood. I was definitely predisposed. My mum has been sick since I was born. She has always struggled with her health. It always felt to me like I was going to lose her*.

*(Sunday Independent, 25/08/2019)*

Other features of one’s family environment included parental divorce, domestic violence, learned behaviours, and high parental expectations (*n* = 15).

*…the emotional anguish she had suffered as a result of her father’s break-up with her stepmother, Orianne, in 2008, triggered the eating disorder anorexia nervosa*

*(Irish Daily Mail, 11/03/2020)*

### Comparison across mental disorders

**Fig 3:**
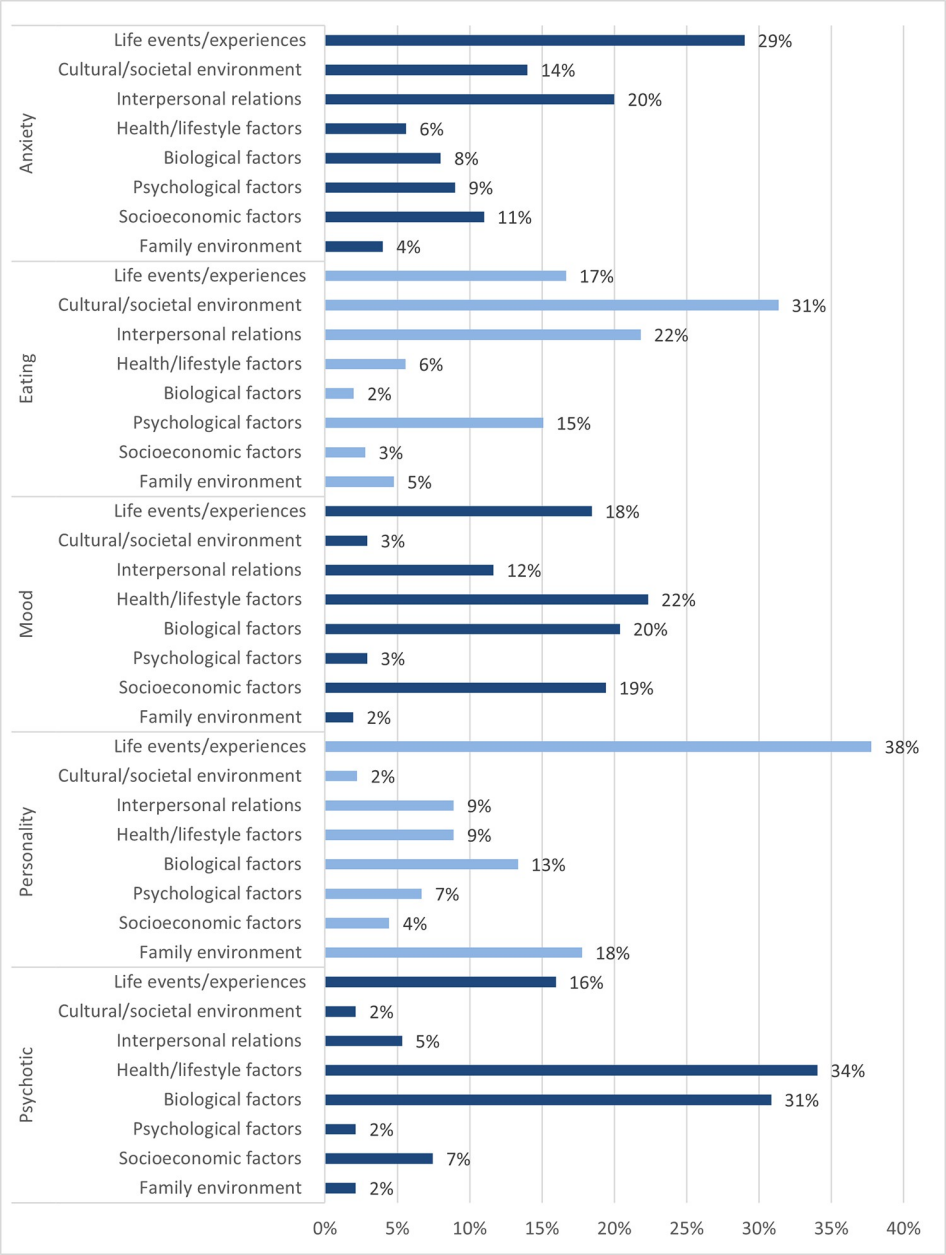
Frequency percentage of causal explanations within each category of mental disorders. Percentage values represent the frequency of each causal explanation for a given mental disorders category, as a percentage of the total number of causal explanations in that category.

An analysis of frequency was conducted which compared the prevalence of the eight identified causal explanations for each of the included mental disorder categories; psychotic disorders, personality disorders, mood disorders, eating disorders, and anxiety disorders (Fig 3). Results pertaining to the substance-related disorders category were omitted due to the low prevalence of articles containing causal explanations (*n* = 2), both of which described health/lifestyle attributions. While the value of this data is primarily descriptive due to challenges in meaningfully interpreting a 5x8 statistical comparison, chi square analysis did confirm a significant association between causal explanations and mental disorders category, *χ*^2^(28, *N* = 637) = 286.55, *p* < .001. Results show that life events/experiences were the most common explanation for anxiety and personality disorders, cultural/societal environment for eating disorders, and health/lifestyle factors for mood and psychotic disorders. Biological factors were most invoked for psychotic or mood disorders, and appeared infrequently in relation to anxiety, eating or personality disorders.

### Frequency comparison before and during the COVID-19 pandemic

**Fig 4:**
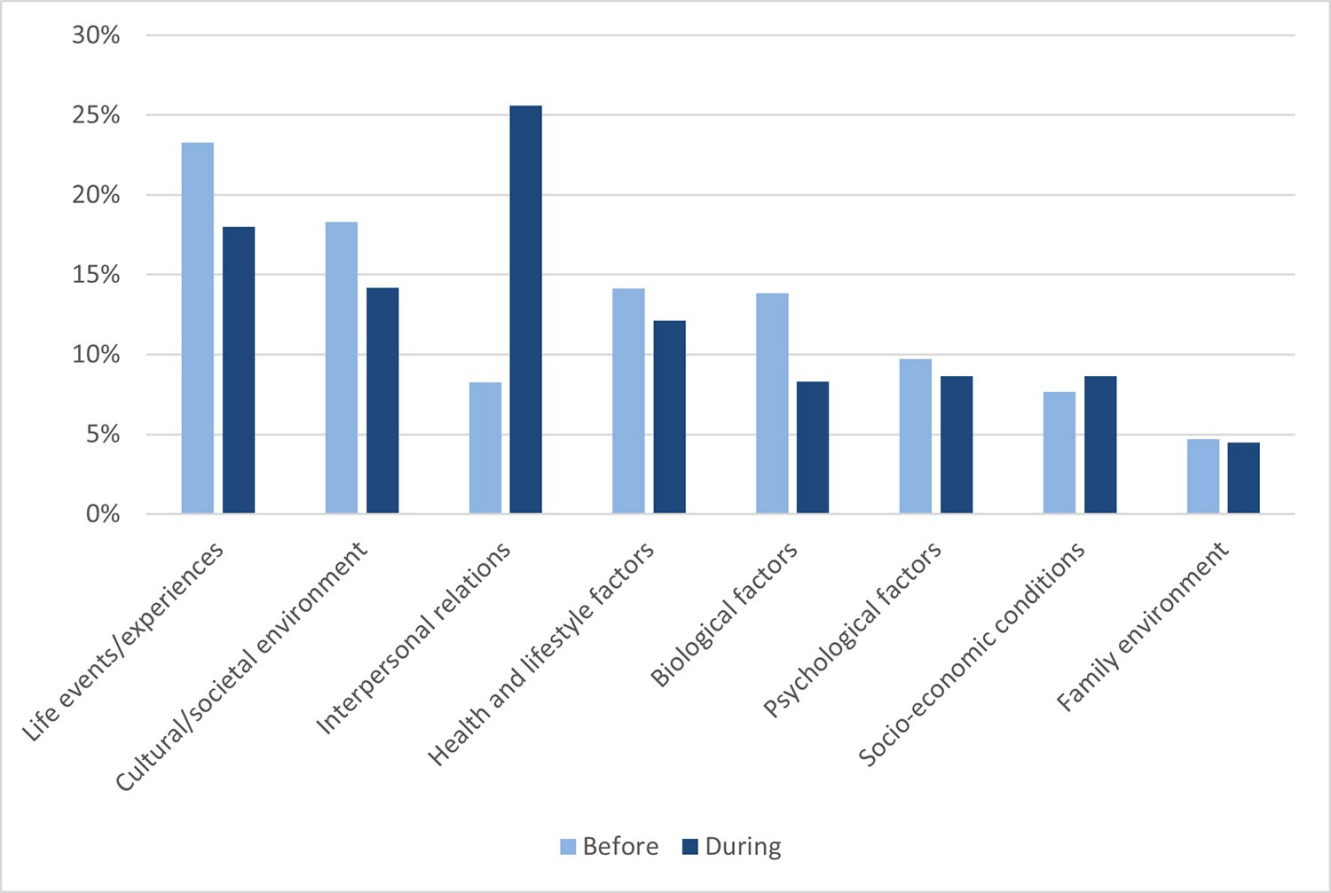
Frequencies of causal explanations present in Irish news media before and during the COVID-19 pandemic. Before: 12^th^ March 2018 -11^th^ March 2020 (*n* = 339). During: 12^th^ March 2020 – 12^th^ Match 2022 (*n* = 289). Percentage values represent the frequency at which attributions occurred as a percentage of the total number of attributions in that time period.

To assess whether there were observable differences in representations of causal explanations before and during the COVID-19 pandemic, a frequency analysis was conducted (Fig 4). Overall, there were slightly fewer causal explanations in news media during the pandemic (*n* = 289) than before (*n* = 339). A chi square analysis revealed that the distribution of causal explanations differed significantly before and during the pandemic, *χ*^2^(7, *N* = 628) = 37.77, *p* < .001. To pinpoint the specific categories responsible for this difference, eight 2x2 post-hoc analyses using a Bonferroni correction were conducted. This revealed a significant effect (*p* < .006) only within the interpersonal relations category, which rose from 8.62% of attributions before the pandemic, to 26% of attributions during the pandemic.

## Discussion

While causal attributions have been a sustained focus of efforts to understand the stigma dynamics of mental disorders, minimal research has investigated how causal explanations naturally surface in everyday discourse. The current study provides original insight into the range, relative prevalence, and contextual variability of naturally occurring causal attributions for mental disorders.

### How are causal explanations for mental disorders represented in Irish news media?

Content analysis of over 400 news articles identified eight superordinate types of explanation for mental disorders in media discourse: life events/experiences, the cultural/societal environment, interpersonal relations, health/lifestyle factors, biological factors, psychological factors, socio-economic conditions, and the family environment. Overall, the most commonly occurring explanations involved attributions to social phenomena, such as life events/experiences, the cultural/societal environment, and interpersonal relations. Conversely, biological factors accounted for just over one-tenth of observed causal attributions. This arguably indicates the relative volume of interest in biological attributions in previous research may be disproportionate to their real-world frequency within naturally occurring public discourse.

As social attributions are much more frequent in real-world discourse about mental disorders than currently represented in attribution research, they merit more direct theoretical and empirical elaboration on their own terms, rather than as merely a foil or control for biological attributions. While most previous research has collapsed social explanations into a single unidimensional category, the current data illuminates the multi-layered nature of attributions to social causes. Articles invoked determinants that traversed multiple ‘levels’ of the social world: from individual biographies to interpersonal relations, family dynamics, socio-economic factors and cultural context. Research from other domains suggests that different social determinants can trigger variable degrees of social essentialism, and hence more negative intergroup attitudes [22]. While investigating the differential implications of divergent social attributions was beyond the scope of this study, the analysis provides proof-of-concept of substantial variability within the content of social explanations and emphasises the need for future research to take account of this nuance.

The high prevalence of social explanations overall in comparison to biological factors is consistent with some previously documented survey data, which imply that people most commonly attribute mental disorders to psychosocial factors, rather than biological [5]. However, previous studies have found personal factors comprise a large proportion of commonly-attributed ‘psychosocial’ factors [5]; in the current study, psychological factors accounted for only 8% of the present explanations. This difference may be a methodological artefact. Previous research regarding public aetiological beliefs has typically administered surveys containing a restrictive range of options from which participants may respond [5]. This can obscure attributions that the researchers have not anticipated or spotlight attributions that participants would not spontaneously volunteer–hence the importance of observing naturally occurring discourse. Of course, even naturalistic datasets are subject to the unique contingencies of that form of communication. For example, the infrequent reference to psychological causes in media data may reflect writers’ tendencies to avoid introducing causal explanations that imply a high degree of personal blame towards the affected person, in an attempt to ameliorate sigma narratives. Additionally, the position of life events/experiences as the most frequent attribution overall (20.86%) may be a result of news sources placing greater focus on reporting recent events of significance, such as crimes or serious accidents. Analysis of more informal naturalistic data, such as social media or verbal conversations, may yield different results.

The relatively low prevalence of biological explanations is notable. It suggests that concerns about the rising societal prominence of biomedical understandings of mental ill-health may be premature, at least within this cultural context. However it is worth noting that in the present findings, health/lifestyle factors presented similar patterns to biological factors in terms of their distributions across mental disorder categories. Features of this attribution category included factors such as substances/medication, lack of sleep, health issues, and diet/malnourishment. As these are primarily physical in nature, it is possible they are conceptualised in a similar manner to biological factors, though further research is needed to investigate this.

### Are causal explanations represented differently across different categories of mental disorders?

While the overall findings presented higher rates of social attributions, this was not universal across mental disorder categories. Indeed, the overall figures disproportionately reflect the mental disorder categories which contained more causal explanations, such as eating disorders. This reiterates the importance of focusing analysis at the level of specific mental disorder categories, rather than a generic construct of ‘mental illness’. While eating disorders and anxiety disorders were most commonly attributed to social factors such as the cultural/societal environment, interpersonal factors, and life events/experiences, psychotic and mood disorders were most commonly attributed to health/lifestyle or biological factors. Additionally, while personality disorders were most commonly attributed to life events/experiences, they generated comparatively frequent rates of health/lifestyle and biological attributions.

These trends present both similarities and differences to previously documented patterns in public perceptions of aetiology. In terms of similarities, the findings reflect public tendencies to attribute personality disorders to trauma and stress [39, 40], and to more frequently endorse biological determinants for schizophrenia than other mental disorders [23]. However, the present findings differ from research which suggests that eating disorders are more likely to be attributed to personal factors [41], and that depression and schizophrenia are most likely to be attributed to acute stress [42]. Again, these differences may reflect divergence between media and survey methodologies; further research is required to clarify.

Because of the stigma implications of public perceptions of aetiology, it is also beneficial to consider how the findings map onto previously observed patterns in societal stigma. In the present study, more traditionally stigmatised categories of mental disorders were more likely to be attributed to biological or health/lifestyle factors than less stigmatised categories. Previous research has found that personality and psychotic disorders are highly stigmatised in comparison to other mental disorders types such as eating disorders or anxiety disorders [43]. These observed patterns align with theories of psychological essentialism which imply that biological attributions are associated with greater stigma attitudes, such as perceptions of dangerousness and desire for social distance [1].

### What proportion of articles that mention mental disorders contain causal explanations?

Results indicated that approximately one-quarter of all articles that contained one of the specified diagnostic keywords included an explanation of the causes of that disorder. While it is difficult to interpret this figure without appropriate benchmarks, results confirm that causal attributions spontaneously occur on a regular basis in mental disorders discourse [5]. Given their documented significance for stigma dynamics [1], the study thus validates the maintenance of causal attributions as a research priority.

### Did patterns of causal explanations change during the COVID-19 pandemic?

As a major societal event, the COVID-19 pandemic offers a good test-case to explore the malleability of causal attributions across socio-cultural context. The analysis produced some tentative evidence of temporal changes in attribution patterns post-pandemic, most notably a sharp rise in attributions to interpersonal relations, which included factors such as loneliness and social isolation. Along with prior research [27], this finding suggests that the pandemic may have increased public sensitivity to the social causes of mental disorders, though the durability of such effects remains to be seen. This emphasises the importance of situating research on causal attributions within their cultural/societal environment, and diversifying the geographic and historical evidence-base from which conclusions about attributions’ trends and effects are drawn.

### Strengths and limitations

The study’s original contributions emanate from its observation of naturally occurring discourse, which garnered ecological validity that cannot be generated by artificially-constructed survey data alone. However, there are several limitations to the present study that should be considered. Firstly, it should be noted that public consumption of media is not exclusive to print and online news media sources alone, with notable alternative sources including social media, television, radio, and film. However, a strength to assessing news media specifically is the high level of trust and perceived reliability associated with it in comparison to other sources of information, such as social media [44, 45]. Moreover, much social media content involves dissemination of information originally issued via traditional media channels [46].

Secondly, the search terms in the current study present some limitations. While the use of DSM-5 diagnostic terms allowed for meaningful comparison across mental disorder types, and ensured the dataset was not overwhelmed by non-clinical references to terms such as ‘anxiety’ and ‘addiction’, results were affected by some diagnostic terms being more widely used than others. For example, the terms “anorexia nervosa” and “phobia” were used relatively faithfully, but the number of articles recovered about substance addiction was low due to infrequent lay use of formal diagnostic terms like “alcohol use disorder”. Similarly, the mood disorder category was more representative of bipolar disorder than major depressive disorder due to greater commonality of the term “bipolar disorder” than “major depressive disorder” in everyday language. The search terms also did not capture generic discussions of “mental health” or “mental illness”. While the analysis did not aim or claim to cover every single article published on the topic, it is unclear whether these keyword contingencies may have systematically biased the dataset in some way. The analysis itself sought to guard against bias through using an inductive code-development strategy and inter-coder reliability assessment.

Thirdly, the findings are necessarily limited to one particular cultural context, in a unique historical moment. This in itself represents an original contribution, as minimal published research has investigated Irish patterns of explaining mental disorders, or pandemic-related effects on attribution internationally. It is unclear whether or how unique features of the Irish Media landscape affect results. For instance, Irish newspapers typically do not have clear affiliations with certain political parties or ideologies. As political affiliations are associated with attributional tendencies, with right-wing attitudes more liked with biological and left-wing with social attributions [47], a more politicised media landscape may yield different results. Further work is required to establish how the patterns detected in this Irish study compare with other cultural contexts.

Finally, results are descriptive and do not facilitate inference about the cause or effects of the attributional patterns demonstrated. However this is appropriate for an initial exploratory study. This descriptive data is vital to inform future ecologically valid, hypothesis-driven research, for example regarding the influence of COVID-19 on attributions or the differential effects of various social attributions.

## Conclusions

The present study pioneered an original investigation of how causal representations for mental disorders spontaneously manifest in naturalistic public discourse. Findings revealed a wide range of attributions for mental disorders present in Irish news media, particularly in the diverse forms of social explanations offered. While social attributions were more common than biological attributions overall, attributions were unevenly distributed across mental disorder category, with more traditionally stigmatised categories, such as psychotic disorders, tending to be more biologized than traditionally less stigmatised categories, such as anxiety disorders. The COVID-19 pandemic also coincided with a shift in attribution patters, most notably a rise in attributions to interpersonal relations. The study proposes several avenues for progressing research on causal explanations of mental disorders: most notably more incorporation of naturally occurring data, investigation of the full range of social attributions and their distinct attitudinal effects, clarification of how attributional patterns diverge across different forms of mental disorders, and greater attention to cultural context and change over time.

## Supporting information

S1 Table Superordinate and basic codes and their corresponding frequencies and levels of inter-coder agreement. (DOCX)

S2 Table Included news sources (print and online). (DOCX)

S1 Appendix Search terms. (DOCX)

## References

[1] Haslam N, Kvaale EP. Biogenetic explanations of mental disorder: The mixed-blessings model. Curr Dir Psychol Sci. 2015 Oct 1;24(5):399–404.

[2] Lebowitz MS, Appelbaum PS. Biomedical explanations of psychopathology and their implications for attitudes and beliefs about mental disorders. Annu Rev Clin Psychol. 2019;15(1):555–77. doi: 10.1146/annurev-clinpsy-050718-095416 30444641 PMC6506347

[3] McCombs M, Valenzuela S. Setting the agenda: Mass media and public opinion. John Wiley & Sons; 2020. 232 p.

[4] Angermeyer MC, Dietrich S, Pott D, Matschinger H. Media consumption and desire for social distance towards people with schizophrenia. Eur Psychiatry J Assoc Eur Psychiatr. 2005 May;20(3):246–50. doi: 10.1016/j.eurpsy.2004.12.005 15935424

[5] Furnham A, Swami V. Mental health literacy: A review of what it is and why it matters. Int Perspect Psychol Res Pract Consult. 2018;7:240–57.

[6] Weiner B. On sin versus sickness: A theory of perceived responsibility and social motivation. Am Psychol. 1993;48:957–65. 8214914 10.1037//0003-066x.48.9.957

[7] Manusov V, Spitzberg B. Attribution theory: Finding good cause in the search for theory. In: Engaging Theories in Interpersonal Communication: Multiple Perspectives [Internet]. 2455 Teller Road, Thousand Oaks California 91320 United States: SAGE Publications, Inc.; 2008. p. 37–50. Available from: https://sk.sagepub.com/books/engaging-theories-in-interpersonal-communication/n3.xml

[8] Sayre J. The patient’s diagnosis: Explanatory models of mental illness. Qual Health Res. 2000 Jan 1;10(1):71–83. doi: 10.1177/104973200129118255 10724754

[9] McCormack L, Thomson S. Complex trauma in childhood, a psychiatric diagnosis in adulthood: Making meaning of a double-edged phenomenon. Psychol Trauma Theory Res Pract Policy. 2017;9:156–65. doi: 10.1037/tra0000193 27710004

[10] Baines T, Wittkowski A. A Systematic Review of the Literature Exploring Illness Perceptions in Mental Health Utilising the Self-Regulation Model. J Clin Psychol Med Settings. 2013 Sep 1;20(3):263–74. doi: 10.1007/s10880-012-9337-9 23108509

[11] Read J, Haslam N, Sayce L, Davies E. Prejudice and schizophrenia: a review of the ‘mental illness is an illness like any other’ approach. Acta Psychiatr Scand. 2006;114(5):303–18. doi: 10.1111/j.1600-0447.2006.00824.x 17022790

[12] Schomerus G, Schwahn C, Holzinger A, Corrigan PW, Grabe HJ, Carta MG, et al. Evolution of public attitudes about mental illness: a systematic review and meta-analysis. Acta Psychiatr Scand. 2012;125(6):440–52. doi: 10.1111/j.1600-0447.2012.01826.x 22242976

[13] Huggard L, Murphy R, O’Connor C, Nearchou F. The social determinants of mental illness: A rapid review of systematic reviews. Issues Ment Health Nurs. in press; 10.1080/01612840.2023.2186124 36972547

[14] Haslam SA, McMahon C, Cruwys T, Haslam C, Jetten J, Steffens NK. Social cure, what social cure? The propensity to underestimate the importance of social factors for health. Soc Sci Med. 2018 Feb 1;198:14–21. doi: 10.1016/j.socscimed.2017.12.020 29274614

[15] Read J, Mosher LR, Bentall RP. Models of madness: psychological, social and biological approaches to schizophrenia. Psychology Press; 2004. 398 p.

[16] Schroder HS, Duda JM, Christensen K, Beard C, Björgvinsson T. Stressors and chemical imbalances: Beliefs about the causes of depression in an acute psychiatric treatment sample. J Affect Disord. 2020 Nov 1;276:537–45. doi: 10.1016/j.jad.2020.07.061 32807732

[17] Pescosolido BA, Manago B, Monahan J. Evolving public views on the likelihood of violence from people with mental illness: Stigma and its consequences. Health Aff (Millwood). 2019 Oct;38(10):1735–43. doi: 10.1377/hlthaff.2019.00702 31589533

[18] Schomerus G, Schindler S, Sander C, Baumann E, Angermeyer MC. Changes in mental illness stigma over 30 years–Improvement, persistence, or deterioration? Eur Psychiatry. 2022 ed;65(1):e78. 36328960 10.1192/j.eurpsy.2022.2337 PMC9724218

[19] Dittrich D, Dernbach K, Speerforck S, Schindler S, Häusser JA, Schomerus G. Testing the mixed-blessings model: What is the role of essentialism for stigmatizing attitudes towards schizophrenia? Curr Psychol [Internet]. 2021 Mar 30 [cited 2022 Jul 18]; Available from: 10.1007/s12144-021-01653-3

[20] Kvaale EP, Gottdiener WH, Haslam N. Biogenetic explanations and stigma: A meta-analytic review of associations among laypeople. Soc Sci Med. 2013 Nov 1;96:95–103. doi: 10.1016/j.socscimed.2013.07.017 24034956

[21] Kvaale EP, Haslam N, Gottdiener WH. The ‘side effects’ of medicalization: A meta-analytic review of how biogenetic explanations affect stigma. Clin Psychol Rev. 2013 Aug 1;33(6):782–94. doi: 10.1016/j.cpr.2013.06.002 23831861

[22] Rangel U, Keller J. Essentialism goes social: belief in social determinism as a component of psychological essentialism. J Pers Soc Psychol. 2011 Jun;100(6):1056–78. doi: 10.1037/a0022401 21319911

[23] Angermeyer MC, Dietrich S. Public beliefs about and attitudes towards people with mental illness: a review of population studies. Acta Psychiatr Scand. 2006;113(3):163–79. doi: 10.1111/j.1600-0447.2005.00699.x 16466402

[24] O’Connor C, Brassil M, O’Sullivan S, Seery C, Nearchou F. How does diagnostic labelling affect social responses to people with mental illness? A systematic review of experimental studies using vignette-based designs. J Ment Health Abingdon Engl. 2022 Feb;31(1):115–30. doi: 10.1080/09638237.2021.1922653 34008456

[25] Coey P., Nic Craith I., McQuaid L., D’Alton P., & O’Connor C. Does explaining psychogenic non-epileptic seizures using either a biomedical or biopsychosocial framework affect young people’s illness representations? An experimental vignette study. Epilepsy Behav. in press; 10.1016/j.yebeh.2023.109186 37028150

[26] Furnham A, Hamid A. Mental health literacy in non-western countries: a review of the recent literature. Ment Health Rev J. 2014 Jan 1;19(2):84–98.

[27] O’Connor C. Has the COVID-19 pandemic affected lay beliefs about the cause and course of mental illness? Int J Environ Res Public Health. 2021 Jan;18(9):4912. doi: 10.3390/ijerph18094912 34063004 PMC8124589

[28] Bauer MW. Distinguishing Red and Green Biotechnology: Cultivation Effects of the Elite Press. Int J Public Opin Res. 2005 Mar 1;17(1):63–89.

[29] O’Connor C, Joffe H. The social aetiology of essentialist beliefs. Behav Brain Sci. 2014 Oct;37(5):498–9. doi: 10.1017/S0140525X1300383X 25388046

[30] Wagner W, Kronberger N, Seifert F. Collective symbolic coping with new technology: Knowledge, images and public discourse. Br J Soc Psychol. 2002;41(3):323–43. doi: 10.1348/014466602760344241 12419006

[31] Kelly BD. Hearing Voices: Lessons from the History of Psychiatry in Ireland. Ir Med J. 2017 Mar 1;110(3):537. 28657250

[32] Anonymous. Health at a Glance: Europe [Internet]. Public Health—European Commission. 2016 [cited 2021 Nov 9]. Available from: https://ec.europa.eu/health/state/glance_en

[33] Kelly BD. Impact of COVID-19 on mental health in Ireland: evidence to date. Ir Med J. 2020;113(10):214.

[34] Gauchat G. The cultural authority of science: Public trust and acceptance of organized science. Public Underst Sci. 2011 Nov 1;20(6):751–70. doi: 10.1177/0963662510365246 22397083

[35] American Psychiatric Association American Psychiatric Association. DSM-5 Task Force. Diagnostic and statistical manual of mental disorders: DSM-5 [Internet]. 5th ed. London; Washington, D.C; American Psychiatric Publishing; 2013. Available from: https://go.exlibris.link/XMGlrCnp

[36] Dattani S, Ritchie H, Roser M. Mental Health. Our World Data [Internet]. 2021 Aug 20 [cited 2022 Nov 22]; Available from: https://ourworldindata.org/mental-health

[37] Krippendorff K. Content analysis: An introduction to its methodology. SAGE; 2013. 457 p.

[38] O’Connor C, Joffe H. Intercoder Reliability in Qualitative Research: Debates and Practical Guidelines. Int J Qual Methods. 2020 Jan 1;19:1609406919899220.

[39] Furnham A, Lee V, Kolzeev V. Mental health literacy and borderline personality disorder (BPD): what do the public “make” of those with BPD? Soc Psychiatry Psychiatr Epidemiol. 2015 Feb 1;50(2):317–24. doi: 10.1007/s00127-014-0936-7 25064182 PMC4308651

[40] Wright K, Furnham A. What Is narcissistic personality disorder? Lay theories of narcissism. Psychology [Internet]. 2014 Jul 11;2014. Available from: http://www.scirp.org/journal/PaperInformation.aspx?PaperID=48298

[41] O’Connor C, McNamara N, O’Hara L, McNicholas F. Eating disorder literacy and stigmatising attitudes towards anorexia, bulimia and binge eating disorder among adolescents. Adv Eat Disord. 2016 May 3;4(2):125–40.

[42] Dietrich S, Beck M, Bujantugs B, Kenzine D, Matschinger H, Angermeyer MC. The Relationship Between Public Causal Beliefs and Social Distance Toward Mentally Ill People. Aust N Z J Psychiatry. 2004 May 1;38(5):348–54. doi: 10.1080/j.1440-1614.2004.01363.x 15144513

[43] Feldman DB, Crandall CS. Dimensions of Mental Illness Stigma: What About Mental Illness Causes Social Rejection? J Soc Clin Psychol. 2007 Feb;26(2):137–54.

[44] Newman N, Fletcher R, Schulz A, Andi S, Robertson CT, Nielsen RK. Reuters Institute Digital News Report 2021 [Internet]. 2021 Jun [cited 2023 Feb 10]. Available from: https://papers.ssrn.com/abstract=3873260

[45] Park S, Fisher C, Flew T, Dulleck U. Global Mistrust in News: The Impact of Social Media on Trust. Int J Media Manag. 2020 Apr 2;22(2):83–96.

[46] Chew C, Eysenbach G. Pandemics in the age of Twitter: Content analysis of Tweets during the 2009 H1N1 outbreak. PLOS ONE. 2010 Nov 29;5(11):e14118. doi: 10.1371/journal.pone.0014118 21124761 PMC2993925

[47] Suhay E, Jayaratne TE. Does biology justify ideology? The politics of genetic attribution. Public Opin Q. 2013;77(2):497–521. 26379311 10.1093/poq/nfs049 PMC4567596

