# Supplementary material for: How does the public understand the causes of mental disorders? An analysis of Irish news media before and during the COVID-19 pandemic
Source: PLoS One. 2023 Apr 6;18(4):e0284095. doi: 10.1371/journal.pone.0284095 (PMC10079019; doi:10.1371/journal.pone.0284095)
Supplement: S1 Table — (DOCX) [file pone.0284095.s001.docx]

**S1 Table: Superordinate and basic codes and their corresponding frequencies and levels of inter-coder agreement.**

| **Causal attribution** | **Frequency** | **Kappa coefficient** |
| --- | --- | --- |
| **Life events/ experiences** | 131 (20.86%) | 0.85 |
| *Traumatic experiences and adversity* | 81 | 0.90 |
| *Grief, losing a loved one* | 16 | 0.96 |
| *Educational pressures* | 13 | 0.99 |
| *Stressful experiences* | 12 | 0.94 |
| *Environmental factors (unspecified)* | 6 | 0.96 |
| *Pregnancy/childbirth* | 5 | 0.98 |
| *Other life events/experiences* | 2 | 0.93 |
| **Cultural/societal environment** | 103 (16.40%) | 0.91 |
| *Cultural/societal expectations* | 56 | 0.91 |
| *Social media/the internet* | 32 | 0.98 |
| *Exposure to fear-inducing or distressing information* | 11 | 0.98 |
| *Other cultural/societal environment factors* | 9 | 0.94 |
| ***Interpersonal relations*** | 102 (16.24%) | 0.88 |
| *Social isolation, loneliness and COVID-19 related lockdowns* | 50 | 0.96 |
| *Maltreatment* | 45 | 0.92 |
| *Contact with other people with mental disorders* | 2 | 0.99 |
| *Other interpersonal relations* | 7 | 0.96 |
| ***Health/lifestyle factors*** | 83 (13.22%) | 0.90 |
| *Effects of substances and medications* | 38 | 0.99 |
| *Diet* | 17 | 0.98 |
| *Sleep* | 13 | - |
| *Physical disorders/health issues* | 7 | 0.99 |
| *Other health and lifestyle factors* | 8 | 0.95 |
| ***Biological factors*** | 71 (11.30%) | 0.94 |
| *Genetics* | 41 | 0.95 |
| *Hormones and neurotransmitters* | 10 | 0.99 |
| *Brain injury* | 3 | 0.99 |
| *Other biological factors* | 22 | 0.94 |
| ***Psychological factors*** | 58 (9.24%) | 0.89 |
| *Emotional self-management* | 26 | 0.92 |
| *Personality, disposition, or character* | 23 | 0.96 |
| *Other psychological factors* | 9 | 0.93 |
| ***Socio-economic conditions*** | 51 (8.12%) | 0.94 |
| *Employment conditions* | 23 | 0.97 |
| *Financial strain, poverty* | 13 | 0.99 |
| *Housing and living conditions* | 11 | 0.96 |
| *Other socio-economic conditions* | 6 | 0.99 |
| ***Family environment*** | 29 (4.62%) | 0.89 |
| *Lack of parental attention or affection* | 8 | 0.97 |
| *Having a parent with physical or mental health issues* | 6 | 0.94 |
| *Other family environment factors* | 15 | 0.93 |
