# Supplementary material for: How does the public understand the causes of mental disorders? An analysis of Irish news media before and during the COVID-19 pandemic
Source: PLoS One. 2023 Apr 6;18(4):e0284095. doi: 10.1371/journal.pone.0284095 (PMC10079019; doi:10.1371/journal.pone.0284095)
Supplement: S2 Table — (DOCX) [file pone.0284095.s002.docx]

**S3 Table: Included News Sources (print and online).**

| **News source** |
| --- |
| Sunday Tribune |
| Sunday Business Post |
| Sunday Independent (Ireland) |
| RTE News |
| Metro Herald (Ireland) |
| The Irish Times |
| The Irish Post |
| Irish Independent |
| Irish Examiner |
| Irish Daily Mail |
| BreakingNews.ie |
