# Supplementary material for: How does the public understand the causes of mental disorders? An analysis of Irish news media before and during the COVID-19 pandemic
Source: PLoS One. 2023 Apr 6;18(4):e0284095. doi: 10.1371/journal.pone.0284095 (PMC10079019; doi:10.1371/journal.pone.0284095)
Supplement: S1 Appendix — (DOCX) [file pone.0284095.s003.docx]

**S2 Appendix. Search Terms**

Mental disorder categories were chosen based on global prevalence rates (American Psychiatric Association, 2013; Dattani et al., 2021). Search terms were chosen based on DSM-5 classification terms (American Psychiatric Association, 2013). Classifications that cite cause of onset in their title were excluded (e.g., ‘depressive disorder due to another medical condition’, ‘substance/medication-induced bipolar and related disorder’).

**Personality disorders:**

“personality disorder”

**Mood disorders:**

“depressive disorder” OR (bipolar w/3 disorder) OR (mood w/2 disorder) OR dysthymia OR “dysphoric disorder” OR “cyclothymic disorder”

**Substance-related disorders:**

“substance use disorder” OR “alcohol use disorder” OR “cannabis use disorder” OR “phencyclidine use disorder” OR “hallucinogen use disorder” OR “inhalant use disorder” OR “opioid use disorder” OR “Sedative, Hypnotic, or Anxiolytic Use Disorder” OR “stimulant use disorder” OR “tobacco use disorder”

**Anxiety disorders:**

“anxiety disorder” OR “selective mutism” OR “phobia” OR “panic disorder” OR “agoraphobia”

**Schizophrenia spectrum and other psychotic disorders:**

“schizotypal” OR “delusional disorder” OR “psychotic disorder” OR “schizophreniform disorder” OR “schizophrenia” OR “schizoaffective disorder” OR “catatonia”

**Feeding and eating disorders:**

“pica” OR “rumination disorder” OR “avoidant/restrictive food intake disorder” OR “anorexia” OR “bulimia” OR “binge eating disorder” OR “eating disorder”
